# MicroRNA-183-5p contributes to malignant progression through targeting PDCD4 in human hepatocellular carcinoma

**DOI:** 10.1042/BSR20201761

**Published:** 2020-10-30

**Authors:** Xiaohui Duan, Wei Li, Peng Hu, Bo Jiang, Jianhui Yang, Lixue Zhou, Xianhai Mao, Bingzhang Tian

**Affiliations:** Research Laboratory of Hepatobiliary Tumor, Department of Hepatobiliary Surgery, Hunan Provincial People’s Hospital, The First Affiliated Hospital of Hunan Normal University, Changsha 410005, China

**Keywords:** hepatocellular carcinoma, malignant progression, microRNA-183-5p, PDCD4, poor prognosis

## Abstract

Hepatocellular carcinoma (HCC) remains one of the most common malignant tumors worldwide. The present study aimed to investigate the biological role of microRNA-183-5p (miR-183-5p), a novel tumor-related microRNA (miRNA), in HCC and illuminate the possible molecular mechanisms. The expression patterns of miR-183-5p in clinical samples were characterized using qPCR analysis. Kaplan–Meier survival curve was applied to evaluate the correlation between miR-183-5p expression and overall survival of HCC patients. Effects of miR-183-5p knockdown on HCC cell proliferation, apoptosis, migration and invasion capabilities were determined via Cell Counting Kit-8 (CCK8) assays, flow cytometry, scratch wound healing assays and Transwell invasion assays, respectively. Mouse neoplasm transplantation models were established to assess the effects of miR-183-5p knockdown on tumor growth *in vivo*. Bioinformatics analysis, dual-luciferase reporter assays and rescue assays were performed for mechanistic researches. Results showed that miR-183-5p was highly expressed in tumorous tissues compared with adjacent normal tissues. Elevated miR-183-5p expression correlated with shorter overall survival of HCC patients. Moreover, miR-183-5p knockdown significantly suppressed proliferation, survival, migration and invasion of HCC cells compared with negative control treatment. Consistently, miR-183-5p knockdown restrained tumor growth *in vivo*. Furthermore, programmed cell death factor 4 (PDCD4) was identified as a direct target of miR-183-5p. Additionally, PDCD4 down-regulation was observed to abrogate the inhibitory effects of miR-183-5p knockdown on malignant phenotypes of HCC cells. Collectively, our data suggest that miR-183-5p may exert an oncogenic role in HCC through directly targeting PDCD4. The current study may offer some new insights into understanding the role of miR-183-5p in HCC.

## Introduction

Hepatocellular carcinoma (HCC) ranks as one of the most prevalent and life-threatening malignancies among the population, accounting for approximately 1 million tumor-related deaths worldwide [1–3]. It is estimated that nearly 30000 patients died from HCC in the United States and that approximately 600000 cancer-related deaths are ascribed to HCC worldwide annually [4–7]. Despite the fact that great progress has been made in the diagnosis and therapy of HCC, the prognosis of HCC patients remains quite unsatisfactory [8,9]. It is widely accepted that the high mortality rate of HCC patients may be largely attributed to lack of effective treatment strategies [10,11]. Hence, it is quite necessary to generate novel therapy strategies for HCC patients and gain a comprehensive understanding of HCC development.

It is well documented that microRNAs (miRNAs) are a large class of conserved non-coding RNA molecules containing roughly 22 nucleotides [12–14]. An increasing number of scientific evidence have demonstrated that miRNAs could exert their biological roles through regulating target gene expression in a post-transcriptional manner [15–17]. To be specific, miRNAs could bind to the 3′ untranslated region (3′UTR) of their target messenger RNA (mRNA) to trigger inhibition of protein translation process [18,19]. In recent decades, mounting studies have demonstrated that miRNAs function as crucial players during the malignant progression of diverse types of human malignant tumors [20–22]. Furthermore, past researches have shown that aberrant expression of miRNAs would contribute to malignant development of human HCC [23–25]. MicroRNA-183-5p (miR-183-5p), a novel cancer-related miRNA, has been identified to act as a critical player in multiple types of human malignancies, including pancreatic adenocarcinoma [26], prostate cancer [27], colorectal cancer [28,29], renal cell cancer [30] and non-small cell lung cancer [31]; nevertheless, its biological role in HCC remains largely unclear.

Programmed cell death factor 4 (PDCD4) belongs to the PDCD protein family [32,33]. It is well acknowledged that the PDCD protein family is involved in a wide range of biological processes, such as cell proliferation, cell survival and cell motility [34,35]. This study aimed to explore the biological role of miR-183-5p in HCC and clarify the potential molecular mechanism involved. In the current study, we characterized the expression patterns of miR-183-5p in clinical tissue samples of HCC patients and evaluated the effects of miR-183-5p knockdown on malignant phenotypes of HCC cells. Taken together, the present study may facilitate a better understanding of miR-183-5p in HCC and provide some new clues for HCC treatment.

## Materials and methods

### Clinical tissue samples

Fifty tumorous tissue samples and paired normal tissue samples were collected from HCC patients who received surgical resection between March 2012 and November 2016. None of these HCC patients underwent chemotherapy, radiotherapy or immunotherapy. Clinical tissue specimen collection was approved by the Ethics Committee of Hunan Provincial People’s Hospital. All the patients gave their written informed consent. The clinical tissue specimens were stored at −80°C for subsequent analysis.

### Cell culture and transfection

Normal human liver L02 cells and the HCC cell lines (Huh-6, Huh-7, SNU-449 and Li-7) were purchased from the Institute of Biochemistry and Cell Biology of the Chinese Academy of Sciences (Shanghai, China). All the cells were cultured in DMEM medium (Gibco, Grand Island, NY, U.S.A.) containing 10% fetal bovine serum (Gibco). Cell transfection was performed using Lipofectamine 2000 (Invitrogen, Carlsbad, CA, U.S.A.) according to the manufacturer’s instructions. Negative control inhibitor (NC inhibitor), miR-183-5p inhibitor, miR-183-5p mimics and small interfering RNA specifically targeting PDCD4 (si-PDCD4) were synthesized by GenePharma Co. Ltd (Shanghai, China). When cells were allowed to grow to 70–90% confluence, cell transfection was performed. NC inhibitor or miR-183-5p inhibitor at a final concentration of 50 nM was transfected into HCC cells. For the rescue assay, si-PDCD4 at a final concentration of 50 nM was transfected into miR-183-5p inhibitor-treated HCC cells. The sequence information of NC inhibitor, miR-183-5p inhibitor, si-PDCD4, negative control mimics and miR-183-5p mimics was listed as follows: NC inhibitor, 5′-UUCUCCGAUCUGGCUACAGU-3′; miR-183-5p inhibitor, 5′-UUCUGACCAUCUUAAGUGAU-3′; si-PDCD4, 5′-GGAGGUGGAUGUGAAAGAU-3′; negative control mimics, 5′-ACUACUGAGUGACAGUAGA-3′; miR-183-5p mimics, 5′-UAUGGCACUGGUAGAAUUCACU-′3.

### Cell proliferation analysis

Cell proliferation was assessed via Cell Counting Kit-8 (CCK8; Dojindo, Tokyo, Japan) in accordance with the manufacturer’s protocol. In brief, approximately 1 × 10^3^ cells per well were seeded into 96-well plates and were cultured at 37°C in a 5% CO_2_ incubator. Then, 20 μl of CCK8 solution was added into each well of 96-well plates and was incubated for another 2 h. The spectrometric absorbance was measured at 450 nm using a microplate reader (Bio-Tek, Winooski, VT, U.S.A.). Each experiment was carried out in triplicates.

### Cell apoptosis analysis via flow cytometry

Cell apoptosis was determined using the Annexin V-FITC/ propidium iodide (PI) cell apoptosis detection kit (Sigma–Aldrich, St. Louis, MO, U.S.A.) according to the manufacturer’s instructions. Briefly, cells were seeded into 24-well plates at a concentration of 1 × 10^6^ cells per well and were incubated with FITC-conjugated Annexin V and PI in the dark for 30 min. Finally, apoptotic cells were determined via a flow cytometer (BD Bioscience, San Jose, CA, U.S.A.).

### DAPI/PI double staining

DAPI (4′,6-diamidino-2-phenylindole)/PI double staining was used to accomplish microscopic observation of apoptotic cells. In brief, 20 μl of staining solution containing DAPI (5 μg/ml) and PI (3 μg/ml) was placed on the slide-mounted cell samples without any fixation treatment. Subsequently, the cell samples were incubated in the dark for 30 min at room temperature. In the end, the samples were observed and photographed under a phase contrast and fluorescence microscopy (Olympus BX60; Olympus, Tokyo, Japan).

### Scratch wound healing assays

Scratch wound healing assays were performed to evaluate migration capability of HCC cells. In brief, cells were seeded into six-well plates at a density of 1 × 10^5^ cells/well. The cells were allowed to grow to ∼80% confluence, and the scratch wound was generated using a 200-μl pipette tip. Subsequently, the scratched cells were cultured for 24 h and observed under an inverted microscope (Olympus). The migration ability was assessed based on the scratch wound healing distances.

### Transwell invasion assays

For invasion ability evaluation, approximately 5 × 10^4^ cells were plated in the top chambers with a Matrigel-coated membrane (BD Bioscience, San Jose, CA, U.S.A.) containing serum-free DMEM medium. The bottom chambers were filled with DMEM medium containing 10% fetal bovine serum. After 48-h incubation, the invaded cells were then fixed with 4% *para*-formaldehyde and were stained with Crystal Violet. Finally, the stained cells were counted under a light microscope from five random visual fields.

### RNA extraction and quantitative real-time PCR analysis

Total RNA was extracted using the TRIzol reagent (Invitrogen) on the basis of the manufacturer’s protocol. For mRNA expression analysis, cRNA was reverse-transcribed using PrimerScript RT Master Mix (Applied Biosystem, Foster, CA, U.S.A.). For miRNA expression analysis, TaqMan miRNA assay kit was applied to evaluate miR-183-5p levels. The quantitative PCR was carried out on an ABI Prism 7500 Fast Real-time PCR system (Applied Biosystems). β-actin was used as an internal control for PDCD4, and U6 served as an internal control for miR-183-5p. In the present study, the 2^−ΔΔ*C*_t_^ method was used to calculate relative RNA expression levels. The sequence information of specific primers was described as followed: miR-183-5p, forward 5′-CGCGGTATGGCACTGGTAGA-3′ and reverse 5′-AGTGCAGGGTCCGAGGTATTC-3′; PDCD4, forward 5′-TGGATTAACTGTGCCAACCA-3′ and reverse 5′-TCTCAAATGCCCTTTCATCC-3′; U6, forward 5′-GCTTCGGCAGCACATATACTT-3′ and reverse 5′-CGCTTCACGAATTTGCGTGT-3′; β-actin, forward 5′-CATGGAGTCCTGTGGCATC-3′ and reverse 5′-CAGGGCAGTGATCTCCTTCT-3′.

### Western blotting analysis

The cellular protein was extracted using RIPA lysis buffer supplemented with proteinase inhibitor (KeyGen, Nanjing, China). Briefly, 20 μg of protein samples were separated using SDS/PAGE and then transferred on to PVDF membranes (Millipore, Billerica, MA, U.S.A.). The PVDF membranes were incubated with primary antibodies overnight at 4°C, including anti-PDCD4 (ab51495; Abcam, Cambridge, MA, U.S.A.) and anti-β-actin (ab8224; Abcam). Subsequently, the PVDF membranes were incubated with horseradish peroxidase-conjugated secondary antibody for 1 h at 37°C. The bands were developed using ECL Western Blotting Detection Kit (Thermo Fisher, Waltham, MA, U.S.A.).

### Luciferase reporter assays

Luciferase reporter assays were conducted to verify the direct binding of miR-183-5p to 3′UTR of PDCD4 mRNA as previously described. In brief, the dual-luciferase expression vectors were constructed via cloning wildtype or mutant PDCD4 mRNA 3′UTR fragments into the pGL3-basic (Promega, Madison, WI, U.S.A.). Subsequently, HEK293T cells were cotransfected with miR-183-5p and wildtype or mutant PDCD4 mRNA 3′UTR. After 48-h incubation, the luciferase activity was measured using a Dual Luciferase Reporter Assay System (Promega). The *Renilla* luciferase was used as a control to normalize the activity of *Firefly* luciferase.

### Tumor transplantation in nude mice

Male BALB/c nude mice (6 weeks of age, *n*=10) were purchased from Shanghai SLAC Laboratory Animal Center (Shanghai, China). The protocols of animal experiments were approved by the Animal Care and Use Committee of Hunan Provincial People’s Hospital. Tumor xenograft models were established through subcutaneous inoculation of approximately 5 × 10^6^ Li-7 cells treated with NC inhibitor or miR-183-5p inhibitor into the flanks of the nude mice. Tumor volumes were measured using slide caliper every 7 days and were calculated through the following formula: volume (mm^3^) = 0.5 × [length (mm) × width^2^ (mm^2^)]. All the mice were euthanized via carbon dioxide inhalation and killed at day 35 post-inoculation.Subsequently, all the neoplasms were removed and weighed.

### Immunohistochemistry analysis

The harvested tumors were stained for immunohistochemical analysis as previously described [36,37]. Briefly, paraffin slides of 4-μm-thickness were deparaffinized and rehydrated. Subsequently, citrate buffer (pH 6.0) was used for antigen retrieval, followed by endogenous peroxidase blocking via 3% hydrogen peroxide treatment. Subsequently, the slides were incubated with the primary antibodies (anti-Ki67, anti-Vimentin and anti-PDCD4) overnight at 4°C, followed by treatment with horseradish peroxidase-conjugated secondary antibody for 30 min. The sections were then stained via diaminobenzidine and counterstained using Hematoxylin. In the end, the sections were observed and photographed under a light microscopy.

### Statistical analysis

SPSS 20.0 software was used to perform statistical analysis. Data were expressed as the mean ± standard deviation. The two-tailed Student’s *t* test was applied to analyze the difference between two groups. One-way analysis of variance (ANOVA) followed by Dunnett’s multiple comparison was used to compare the difference among three groups. The correlation between miR-183-5p expression and clinicopathological features were evaluated using Fisher’s exact test. Pearson’s correlation analysis was carried out to determine the relationship between miR-183-5p expression and PDCD4 mRNA expression in clinical tissue samples. The Kaplan–Meier method was used to analyze the survival curve of HCC patients. A *P*-value of <0.05 was considered statistically significant.

## Results

### Up-regulation of miR-183-5p is observed in HCC

**Figure 1:**
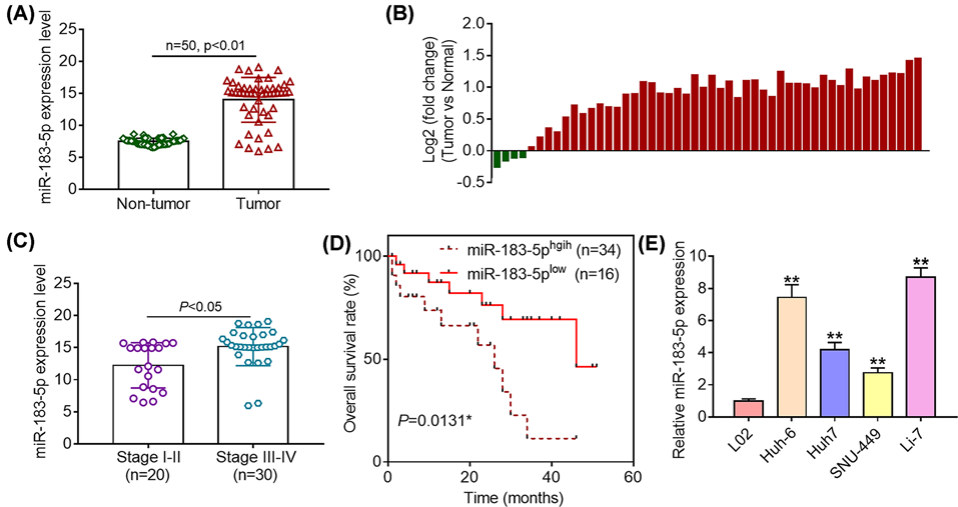
Up-regulation of miR-183-5p is observed in HCC (**A**) miR-183-5p expression was assessed by quantitative RT-PCR analysis in cancerous tissues and adjacent normal tissues from a cohort of HCC patients (*n*=50). (**B**) miR-183-5p was up-regulated in 46 of 50 HCC patients. (**C**) miR-183-5p expression levels were compared by quantitative RT-PCR analysis in tumorous tissues of patients from different TNM stages. (**D**) Overall survival of HCC patients was evaluated using log-rank test and Kaplan–Meier survival analysis. (**E**) miR-183-5p expression was measured in normal human liver L02 cells and HCC cell lines. *P<0.05; **P<0.01. Student’s *t* test was used to compare the difference between two groups. *P<0.05.

To evaluate the biological role of miR-183-5p in HCC, we determined its expression patterns in the cancerous tissues and adjacent normal tissues from a cohort of HCC patients (*n*=50) using quantitative real-time polymerase chain reaction (RT-PCR) analysis. The results showed that a majority of tumorous tissues exhibited higher miR-183-5p expression levels than matched normal tissues (Figure 1A). Furthermore, it was observed that miR-183-5p expression was significantly up-regulated in 46 of 50 HCC patients (Figure 1B). Besides, it was found that cancerous tissues from HCC patients at III–IV stages displayed higher miR-183-5p expression levels than those at I–II stages (Figure 1C). Moreover, we further analyzed the relationship between miR-183-5p expression levels and clinicopathological features of HCC patients. Afterward, HCC patients were divided into high miR-183-5p expression group and low miR-183-5p expression group based on the mean value of its expression levels in tumorous tissues. As shown in Table 1, it was demonstrated that high miR-183-5p expression was closely linked to larger tumor size and advanced TNM stages. Moreover, the Kaplan–Meier survival analysis revealed that HCC patients with elevated miR-183-5p expression experienced a shorter overall survival time (Figure 1D). In addition, we determined the expression of miR-183-5p in normal human liver L02 cells and a panel of HCC cell lines (Huh-6, Huh-7, SNU-449 and Li-7). The results showed that miR-183-5p expression was significantly up-regulated in HCC cell lines compared with that in L02 cells (Figure 1E). Moreover, highest miR-183-5p expression was observed in Huh-6 and Li-7 cell lines. Hence, these two cell lines were selected as cell models to explore the biological functions of miR-183-5p in HCC. To sum up, these lines of evidence indicate that miR-183-5p expression is significantly up-regulated in both HCC tissues and cell lines and that increased miR-183-5p expression correlates with poor prognosis of HCC patients.

**Table 1:** Correlation analysis of miR-183-5p expression and clinicopathological characteristics of HCC patients

Characteristics	Cases	miR-183-5p expression		*P*-value
		Low	High	
Age (years)				
<60	26	9	18	0.308
≧60	24	7	17	
Gender				
Female	27	6	21	0.108
Male	23	10	13	
Lymph node metastasis				
Yes	25	7	18	0.544
No	25	9	16	
Tumor size (cm)				
<5	23	12	11	0.005^†^
≧5	27	4	23	
Liver cirrhosis				
Yes	23	8	15	0.697
No	27	8	19	
TNM stage				
I–II	20	10	10	0.026*
III–IV	30	6	24	
HBV infection				
Yes	26	9	17	0.680
No	24	7	17	

* *P*<0.05.

† *P*<0.01.

### miR-183-5p knockdown suppresses HCC cell proliferation, survival and invasion

**Figure 2:**
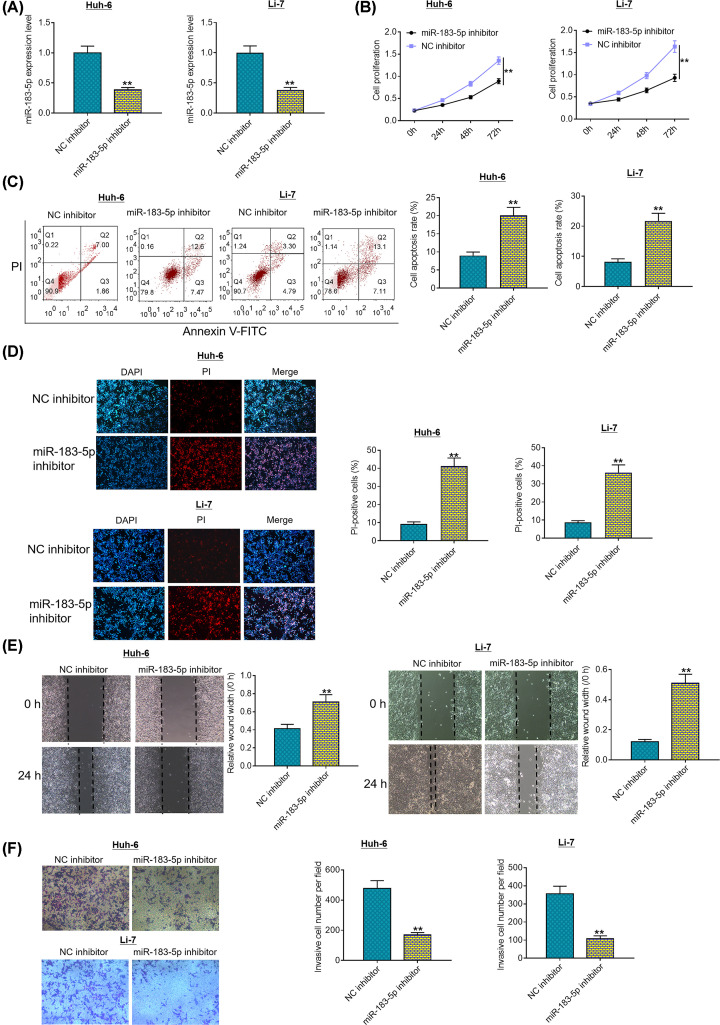
miR-183-5p knockdown suppresses HCC cell proliferation, survival and invasion (**A**) Transfection efficiency in Huh-6 and Li-7 cells was determined using quantitative RT-PCR at 48 h post-transfection with NC inhibitor or miR-183-5p inhibitor. (**B**) Proliferation ability of Huh-6 and Li-7 cells was measured using CCK8 assays after transfection with NC inhibitor or miR-183-5p inhibitor. (**C**) Apoptosis of Huh-6 and Li-7 cells was evaluated by flow cytometry after transfection with NC inhibitor or miR-183-5p inhibitor. (**D**) Apoptosis of Huh-6 and Li-7 cells was visualized via DAPI/PI double staining after transfection with NC inhibitor or miR-183-5p inhibitor. (**E**) Migration ability of Huh-6 and Li-7 cells was assessed by wound healing assays after transfection with NC inhibitor or miR-183-5p inhibitor. (**F**) Invasion capability of Huh-6 and Li-7 cells was determined by Transwell invasion assays after transfection with NC inhibitor or miR-183-5p inhibitor. **P<0.01. Student’s *t* test was used to compare the difference between two groups.

To investigate the potential biological functions of miR-183-5p in HCC, we assessed the effects of miR-183-5p knockdown on malignant phenotypes of Huh-6 and Li-7 cells. First, we analyzed the transfection efficacy of miR-183-5p inhibitor in Huh-6 and Li-7 cells. As presented in Figure 2A, miR-183-5p expression was significantly decreased in miR-183-5p knockdown group compared with that in negative control group. CCK8 assays showed that proliferation abilities of Huh-6 and Li-7 cells were dramatically weakened in miR-183-5p knockdown group compared with negative control group (Figure 2B). Moreover, flow cytometry and DAPI/PI double staining demonstrated a notable increase in apoptotic cells in miR-183-5p knockdown group in comparison with negative control group (Figure 2C,D). As evident from wound healing assays, migration ability of Huh-6 and Li-7 cells decreased by ∼51 and 45% after knockdown of miR-183-5p in comparison with negative control treatment, respectively (Figure 2E). As shown in Figure 2F, Transwell invasion assays revealed that invasion capability of Huh-6 and Li-6 cells decreased by approximately 65 and 70% after knockdown of miR-183-5p in comparison with negative control treatment, respectively. Taken together, these data suggest that miR-183-5p knockdown inhibits HCC cell proliferation, survival and motility.

### PDCD4 is a direct target of miR-183-5p

**Figure 3:**
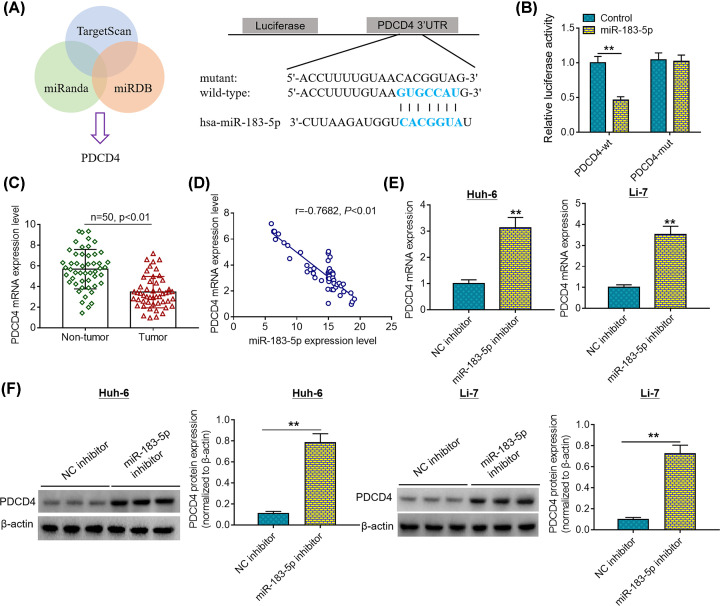
PDCD4 is a direct target of miR-183-5p (**A**) A schematic diagram of the binding site between the seed sequence of miR-183-5p and the 3′UTR of PDCD4 mRNA. (**B**) The relative luciferase activity generated by the luciferase reporter vectors containing wildtype or mutant PDCD4 mRNA 3′UTR was determined examined in 293T cells after transfection of miR-183-5p mimics or negative control mimics. (**C**) PDCD4 mRNA expression levels in cancerous tissues and matched normal tissues of HCC patients (*n*=50) were detected by quantitative RT-PCR analysis. (**D**) The correlation of miR-183-5p expression and PDCD4 mRNA expression was determined by Pearson’s correlation analysis. (**E**) PDCD4 mRNA expression levels were examined by quantitative RT-PCR analysis after transfection with NC inhibitor or miR-183-5p inhibitor. (**F**) PDCD4 protein expression levels were measured by Western blotting analysis. **P<0.01. Student’s *t* test was used to compare the difference between two groups.

To illuminate the potential molecular mechanisms underlying the biological functions of miR-183-5p, bioinformatics analysis was conducted to predict its target genes. Herein, miRanda, miRDB and TargetScan online tools were jointly utilized to forecast the potential targets of miR-183-5p. Among the predicted target genes, PDCD4 elicited our interest for its crucial role in the development of multiple human malignant tumors (Figure 3A). Afterward, luciferase reporter assays were carried out in 293T cells to validate this prediction. As shown in Figure 3B, transfection of miR-183-5p mimics triggered a remarkable decrease in the luciferase activity of the reporter vectors carrying mutant PDCD4 3′UTR fragments, while no significant alterations in the luciferase activity were observed in the reporter vectors carrying mutant PDCD4 3′UTR fragments after transfection of miR-183-5p mimics. Moreover, we detected the expression patterns of PDCD4 in clinical samples using quantitative RT-PCR analysis. It was found that PDCD4 mRNA expression was significantly decreased in tumorous tissues compared with that in adjacent normal tissues (Figure 3C). Furthermore, Pearson’s correlation analysis revealed a negative correlation between miR-183-5p expression and PDCD4 mRNA expression (Figure 3D). Besides, quantitative RT-PCR analysis and Western blotting analysis showed that miR-183-5p inhibitor significantly elevated the mRNA and protein expression levels of PDCD4 in Huh-6 and Li-7 cells compared with negative control group (Figure 3E,F). Collectively, these results indicate that miR-183-5p represses PDCD4 expression through direct binding to its 3′UTR.

### PDCD4 down-regulation alleviates suppressing effects of miR-183-5p knockdown on malignant phenotypes of HCC cells

**Figure 4:**
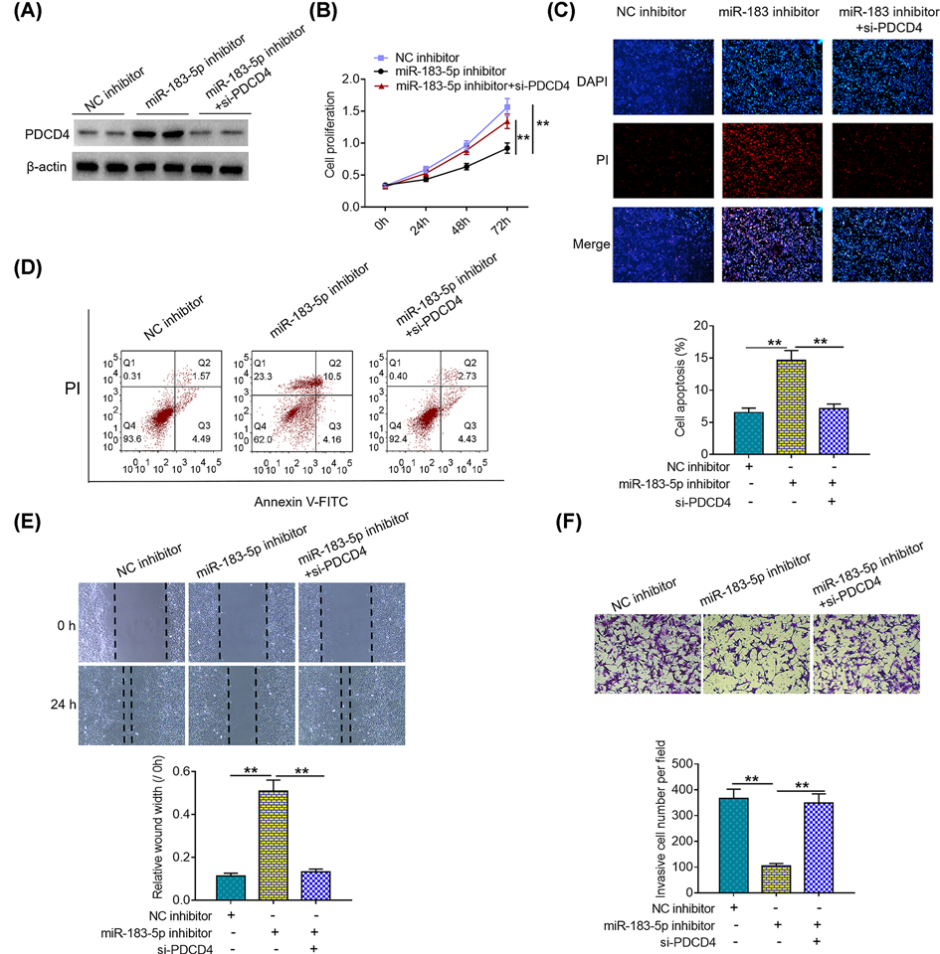
PDCD4 down-regulation alleviates suppressing effects of miR-183-5p knockdown on malignant phenotypes of HCC cells (**A**) PDCD4 protein expression was determined by Western blotting analysis in miR-183-5p inhibitor-treated Li-7 cells after transfection with si-PDCD4. (**B**) Proliferation of miR-183-5p inhibitor-treated Li-7 cells was analyzed via CCK8 assays after transfection with si-PDCD4. (**C**) Apoptosis of miR-183-5p inhibitor-treated Li-7 cells was visualized by DAPI/PI double staining after transfection with si-PDCD4. (**D**) Apoptosis of miR-183-5p inhibitor-treated Li-7 cells was analyzed using flow cytometry after transfection with si-PDCD4. (**E**) Migration capability of miR-183-5p inhibitor-treated Li-7 cells was assessed via wound healing assays after transfection with si-PDCD4. (**F**) Invasion ability of miR-183-5p inhibitor-treated Li-7 cells was detected by Transwell invasion assays after transfection with si-PDCD4. **P<0.01. One-way ANOVA followed by Dunnett’s multiple comparison was used to determine the difference among three different groups.

To better determine the functional relationship between miR-183-5p and PDCD4, we down-regulated PDCD4 expression in miR-183-5p inhibitor-treated Li-7 cells via transfection of si-PDCD4 (Figure 4A). Afterward, we evaluated the effects of PDCD4 down-regulation on malignant phenotypes of miR-183-5p inhibitor-treated Li-7 cells. As presented in Figure 4B–D, PDCD4 down-regulation impaired the inhibitory effects of miR-183-5p inhibitor on Li-7 cell proliferation and survival. Moreover, it was demonstrated that PDCD4 down-regulation abolished the suppressing effects of miR-183-5p inhibitor on migration and invasion capabilities of Li-7 cells (Figure 4E,F). These lines of evidence suggest that miR-183-5p facilitates the malignant phenotypes of HCC cells by directly targeting PDCD4.

### miR-183-5p knockdown inhibits tumor growth in murine xenograft models

**Figure 5:**
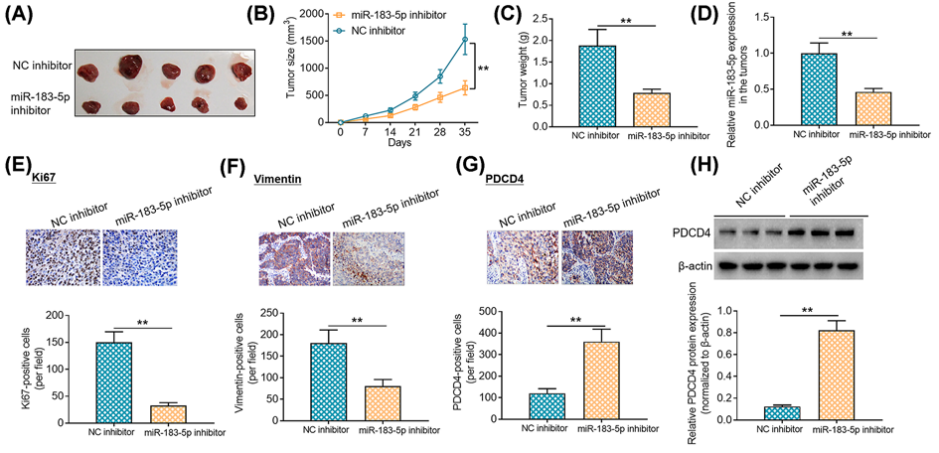
miR-183-5p knockdown inhibits tumor growth in murine xenograft models (**A**) Neoplasm transplantation models were established via subcutaneous injection of Li-7 cells in the flank of BALB/c nude mice (*n*=5). (**B**) The volumes of tumors collected from negative control group and miR-183-5p inhibitor group were measured and recorded every 7 days. (**C**) All the mice were euthanized and killed at day 35 post-inoculation, and the tumors harvested from negative control group and miR-183-5p inhibitor group were weighed. (**D**) Relative expression levels of miR-183-5p. (**E**) Ki67 protein expression was visualized using immunohistochemical staining in the collected tumors. (**F**) Vimentin protein expression was analyzed by immunohistochemical staining in the harvested tumors. (**G**) PDCD4 protein expression was determined via immunohistochemical staining in the collected tumors. (**H**) PDCD4 protein expression in the collected tumors was examined using Western blotting analysis. **P<0.01. Student’s *t* test was used to compare the difference between two groups.

To confirm the findings *in vitro*, neoplasm transplantation models were established in nude mice through subcutaneous injection of Li-7 cells. As exhibited in Figure 5A–C, tumors in miR-183-5p knockdown group grew much slower than those in negative control group; tumors collected from miR-183-5p knockdown group weighed much less than those from negative control group. Moreover, it was found that tumors harvested from miR-183-5p knockdown group exhibited lower miR-183-5p expression than those from negative control group (Figure 5D). Besides, we evaluated the expression patterns of Ki67 and Vimentin proteins in the harvested tumors using immunohistochemical staining. As shown in Figure 5E,F, less Ki67- and Vimentin-positive cells were observed in the tumors harvested from miR-183-5p knockdown group in comparison with negative control group. In addition, immunohistochemical staining and Western blotting analysis showed that tumors from miR-183-5p knockdown group displayed higher PDCD4 protein expression levels than those from negative control group (Figure 5G,H). Taken together, these data imply that miR-183-5p knockdown suppresses tumor growth *in vivo*.

## Discussion

In recent decades, numerous evidence has revealed that miRNAs act as crucial players in the development of various types of human malignancies [38,39]. Accumulating researches have demonstrated that dysregulation of neoplasm-associated miRNAs is involved in the occurrence and development of human HCC [23,24,40]. Thus, identification and characterization of HCC-related miRNAs may contribute to form a better understanding of HCC development and generate effective treatment strategies for HCC patients.

Previous studies have shown that abnormal expression of miR-183-5p is associated with the pathogenesis of multiple kinds of human malignant tumors. Miao et al. reported that miR-183-5p could accelerate pancreatic adenocarcinoma cell proliferation, invasion and metastasis through directly targeting SOCS-6 [26]. Zheng et al. demonstrated that elevated expression of miR-183-5p was observed in colorectal cancer and that miR-183-5p could enhance the radioresistance and promote tumor growth in colorectal cancer via negative regulation of ATG5 [29]. Li et al. found that miR-183-5p expression was up-regulated in renal cell cancer and that up-regulation of miR-183-5p correlated with worse prognosis of patients [30]. Wang et al. revealed that miR-183-5p expression was increased in non-small cell lung cancer and that miR-183-5p promoted tumor growth and metastasis in non-small cell lung cancer by repressing PTEN expression [31].

Except for the oncogenic role in multiple human malignancies mentioned above, miR-183-5p has also been demonstrated to function as a tumor suppressor in acute myeloid leukemia in a recent research [41]. It was reported that miR-183-5p could inhibit occurrence and progression of acute myeloid leukemia through down-regulation of Erbin [41]. As for the discrepancy concerning the biological roles of miR-183-5p in different types of tumors, it may be attributed to the hypothesis that miR-183-5p exhibits tissue-specific expression patterns, thus executing different biological functions in diverse kinds of human neoplasms. In spite of the fact that several previous studies have explored the involvement of miR-183-5p in HCC [42–44], its biological role in HCC and potential molecular mechanism has not been fully understood. Hence, it is quite necessary to further characterize the biological function of miR-183-5p in HCC.

To gain a better understanding of miR-183-5p in HCC, we characterized the expression patterns of miR-183-5p in clinical tissue samples and evaluated the correlation of miR-183-5p expression and overall survival of HCC patients. Our findings demonstrated that miR-183-5p was highly expressed in cancerous tissues in comparison with matched normal tissues and that elevated miR-183-5p expression correlated with shorter overall survival, which was consistent with the reports by Zheng et al. [29], Li et al. [30] and Wang et al. [31]. Thus, the above results imply that miR-183-5p may exert an oncogenic role in HCC. In order to better reveal the biological role of miR-183-5p, we carried out functional researches and evaluated the effects of miR-183-5p depletion on HCC cell proliferation, survival and motility. Our results showed that miR-183-5p knockdown suppressed HCC cell proliferation, facilitated cell apoptosis, and retarded cell migration as well as invasion. Moreover, neoplasm transplantation experiment confirmed that miR-183-5p ablation could repress tumor growth *in vivo.*

In recent years, an increasing number of scientific researches have proposed that miRNAs could exert the biological roles through direct binding to the 3′UTR of their downstream target mRNAs [17,18,21]. In order to clarify the possible molecular mechanisms underlying the oncogenic effects of miR-183-5p in HCC, we carried out further mechanistic studies. Bioinformatics analysis and dual-luciferase reporter assays verified that PDCD4 was a downstream target of miR-183-5p, which was in consistence with previous reports regarding HCC [45,46]. Past researches have identified PDCD4 as a tumor suppressor in HCC [45,46]. Moreover, it was observed that PDCD4 expression was dramatically reduced in HCC tissues compared with paracancerous tissues and that an inverse correlation existed between miR-183-5p and PDCD4 expression. In addition, we conducted rescue assays to evaluate the effects of PDCD4 down-regulation on malignant phenotypes of miR-183-5p inhibitor-treated HCC cells, thereby illuminating the functional correlation between miR-183-5p and PDCD4. It was revealed that PDCD4 down-regulation abrogated the inhibitory effects of miR-183-5p knockdown on HCC cell proliferation, survival and invasion. It is well documented that decreased PDCD4 expression is closely related to the enhanced proliferation, survival and metastasis of tumor cells [47–50].

In summary, the present study for the first time demonstrated that miR-183-5p was highly expressed in HCC and that elevated miR-183-5p expression correlated with poor prognosis. Moreover, our data unveiled that miR-183-5p might exert an oncogenic role in particular by promoting HCC cell proliferation and motility as well as suppressing cell apoptosis. Notably, it was revealed that miR-183-5p could facilitate malignant behaviors of HCC cells through directly targeting PDCD4. Collectively, our findings imply that targeting miR-183-5p/PDCD4 axis may represent a promising intervention strategy for HCC patients. The current study may offer some new insights into understanding the role of miR-183-5p in HCC. Additionally, further researches are still required to validate the biological functions of miR-183-5p in HCC and clarify the detailed action mechanisms in the future studies.

## Abbreviations

CCK8: Cell Counting Kit-8
DAPI: 4′,6-diamidino-2-phenylindole
HCC: hepatocellular carcinoma
miRNA: microRNA
miR-183-5p: microRNA-183-5p
mRNA: messenger RNA
NC inhibitor: negative control inhibitor
PDCD4: programmed cell death factor 4
PI: propidium iodide
RT-PCR: real-time polymerase chain reaction
si-PDCD4: small interfering RNA specially targeting PDCD4
3′UTR: 3′ untranslated region
